# Robotic excision of a large prostatic utricle cyst with microdissection testicular sperm extraction presenting as primary infertility: A case report

**DOI:** 10.1016/j.ijscr.2024.110523

**Published:** 2024-10-26

**Authors:** Michelangelo Cobangbang, Dennis Serrano, Nikko Magsanoc

**Affiliations:** a St. Luke's Medical Center, Institute of Urology, 2nd Floor, Stone and Prostate Treatment Center 279 E. Rodriguez Sr. Blvd., Quezon City, NCR 1102, Philippines; b St. Luke's Medical Center- Institute of Urology, 5th Avenue, Taguig, Metro Manila, NCR 1634, Philippines

**Keywords:** Prostatic utricle cyst, Infertility, Robotic excision, Obstructive azoospermia, Case report

## Abstract

**Introduction and importance:**

Prostatic utricle cyst (PUC) is an anatomic anomaly resulting from a vestigial remnant of the Müllerian duct in the posterior urethra. Proper diagnosis is crucial, as its presentation can vary widely and can present with infertility. The aim of this article is to present a rare case of a large prostatic utricle cyst as a cause of primary infertility, surgically managed with robotic excision followed by microdissection testicular sperm extraction to achieve fertility.

**Case presentation:**

This is a case report of a 29-year-old male presenting with recurrent epididymo-orchitis and primary infertility. Imaging confirmed the presence of an enlarged PUC and was further managed as a case of obstructive azoospermia. Robotic surgery was done owing to its innovative approach in ensuring precise movements, improved anatomical visualization, and finer dissection into the deep pelvic cavity. Successful conception of a healthy offspring was achieved through one attempt of *in vitro* fertilization.

**Clinical discussion:**

Large symptomatic prostatic utricle cysts are managed surgically, whether open, endoscopic, laparoscopic, or robotic. Robotic-assisted laparoscopic excision of prostatic utricle cysts offers many advantages in terms of surgical field exposure, highly improved surgical dexterity and precision, fewer intraoperative complications, and postoperative outcomes. Testicular sperm extraction after surgery is recommended in such cases if fertility is desired.

**Conclusion:**

Robotic excision with microdissection testicular sperm extraction for sperm cryopreservation for future fertility is a feasible and promising option for the treatment of large symptomatic prostatic utricle cysts.

## Highlights

• Prostatic utricle cysts are rare vestigial saccular structures located at the posterior midline of the prostatic urethra.
• Large PUCs may be symptomatic and can present with primary infertility secondary to obstructive azoospermia.
• Robotic excision with mTESE is a feasible and promising option for the treatment of patients with symptomatic PUCs desiring future fertility.
• The entire procedure is a combination of endoscopic, robotic and microscopic surgery to manage a rare clinical entity in urology.

## 1 Introduction

The prostatic utricle is a remnant of the Müllerian duct found in the posterior urethra of males. A prostatic utricle cyst (PUC), on the other hand, is an abnormal dilation or enlargement of the prostatic utricle. There are limited cases on PUC with reported prevalence of 4 % among children and 1 % in adults [1]. When diagnosed, most cases are found concomitantly with other anomalies, such as cryptorchidism, unilateral renal agenesis, and hypospadias [2]. An estimate of 11–14 % are associated with distal hypospadias and disorders of sexual differentiation anomalies [2]. There are also varying presentations between pediatric and adult patients making diagnosis a clinical dilemma. Most cases are asymptomatic and only appear during the first and second decades of life where complications become more troubling to the patient. Complaints are often suggestive of lower urinary tract irritation and obstruction leading to acute urinary retention, post-void dribbling, calculus formation, secondary incontinence, hematospermia, recurrent urinary tract infections, and epididymo-orchitis [3,4]. In rare cases, the presentation of primary infertility from obstructive azoospermia secondary to the PUC has been observed with a reported incidence of approximately 1 % in infertile males [2]. Thus, a high index of suspicion is warranted followed by confirmatory imaging from ultrasonography, retrograde urethrography, magnetic resonance imaging, or direct visualization by cystoscopy.

Large PUCs are anatomically defined as a cyst greater than 1 cm, as PUCs usually measure 0.8 cm to 1.0 cm. However, the size of PUC does not dictate the occurrence of symptoms. In a study by Qiu et al. [5], a PUC of about 2.5 cm in size would lead patients to feel uncomfortable and can show abnormal laboratory findings. This would then warrant further testing and at times, a referral to a urologist. The recurrence and progression of symptoms prompts surgical intervention. However, since PUC is a rare occurrence, various techniques have been applied including open transvesical/perineal approach and laparoscopic excision [3,4]. Even recent surgical developments such as robotics technology is utilized to precisely excise PUC [2,6,7]. In fact, robotic excision may become the technique of choice as it allows better visualization of the relevant anatomy and manipulation of structures ensuring safer and more satisfactory outcomes [6]. Since PUCs are mostly diagnosed in childhood, most case reports are of pediatric patients. Only limited studies identify adult male patients presenting with complications of PUC, with most surgical approaches being laparoscopic or open surgery. This case report presents a 29-year-old male presenting with infertility and recurrent epididymo-orchitis, who was treated with robotic excision of a large prostatic utricle cyst followed by microdissection testicular sperm extraction. The report is prepared in accordance with the Surgical CAse REport (SCARE) Guidelines by the International Journal of Surgery [8].

## 2 Case presentation

**Fig. 1:**
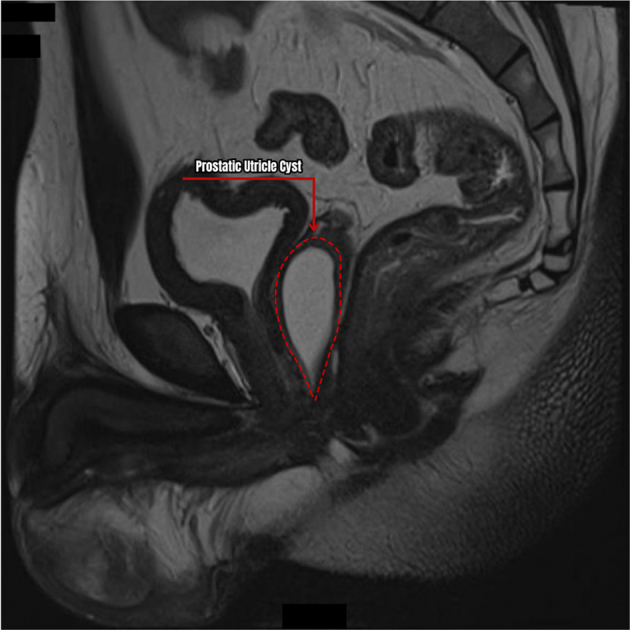
MRI T2-weighted sagittal view of the large prostatic utricle cyst showing its relationship to the rectum and urinary bladder.

A 29-year-old Filipino male with a history of penoscrotal hypospadias, which was surgically corrected in two stages during infancy, had been experiencing recurrent orchitis and urinary tract infections for approximately 10 years. His symptoms included dysuria, incomplete voiding, urinary frequency, and recurrent scrotal fistulae. Initially, the patient responded well to antimicrobial treatment; however, his symptoms recurred frequently. The recurrent symptoms were initially attributed to a penile urethral stricture after hypospadias repair. The stricture was corrected by using a buccal mucosal graft. Despite correction of the stricture, the patient continued to experience recurrent urinary tract infections and orchitis. A lower abdomen magnetic resonance imaging done in 2018 showed a large midline cystic structure occupying the entire prostate gland which measured 1.6 cm × 1.5 cm × 4.4 cm with no discrete focus of restricted diffusion suggestive of a prostatic utricle cyst (Fig. 1). The scrotum was noted to be fibrotic with contents of scar tissue and areas of granulation. There was also bilateral hydrocele most likely secondary to the recurrent infections. The patient was advised to undergo surgical intervention; however, the family opted for maximum conservative management.

One year later, the patient and his 26-year-old female partner were unable to conceive despite adequate attempts. There was no pertinent medical history that predisposes the patient to infertility. Further investigation revealed no family history of infertility, no occupational risk, or socioeconomic background that may contribute to infertility. Physical examination was grossly normal with no scrotal tenderness. On fertility work-up, semen analysis revealed azoospermia. The FSH, LH, and testosterone levels were within normal values at 7.72 mIU/ml, 6.25 mIU/ml, and 8.06 ng/ml, respectively. Diagnostic screening for sexually transmitted infections was negative. Hence, this patient was managed as a case of primary infertility secondary to obstructive azoospermia secondary to prostatic utricle cyst. The patient opted to undergo robot-assisted laparoscopic surgical excision of the PUC. Preoperative laboratory tests were requested, namely sexually transmitted disease workup, infertility workup, and routine laboratory tests (*e.g.* complete blood count, coagulation tests, urinalysis, chest radiograph).

**Fig. 2:**
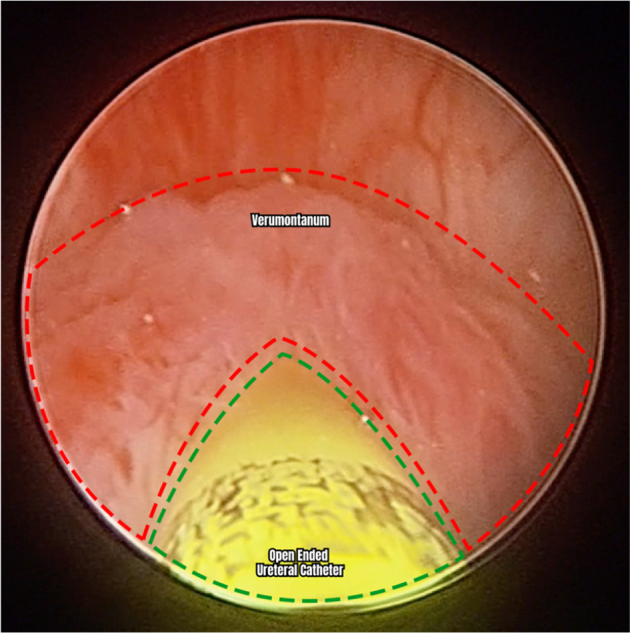
Cystoscopic view of the cannulation of the verumontanum using a 5 French open-ended catheter to access the cavity of the prostatic utricle cyst.

**Fig. 3:**
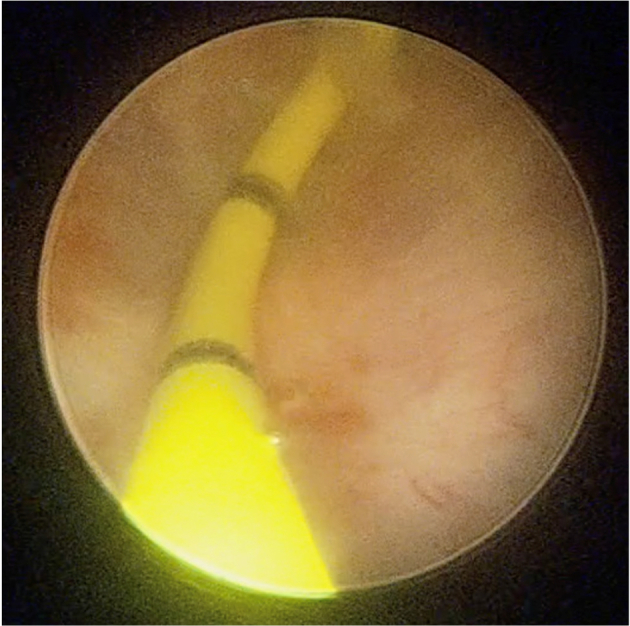
Cystoscopic view of the interior of the PUC after cannulating its opening using a 5 French open-ended catheter.

**Fig. 4:**
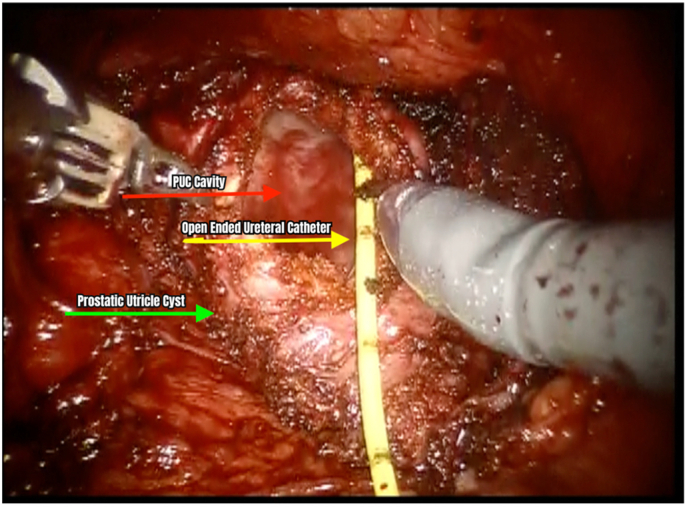
Laparoscopic view of the unroofed prostatic utricle cyst, showing the open-ended ureteral catheter inserted during cystoscopy.

**Fig. 5:**
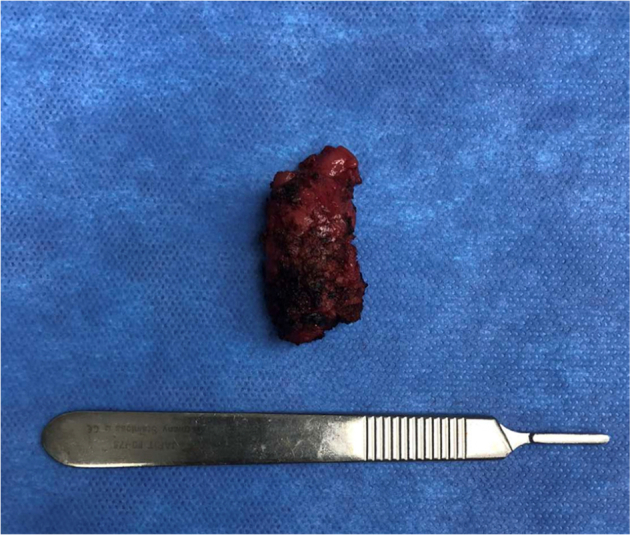
Photo of the excised prostatic utricle cyst.

On the day of operation, the patient was placed on general anesthesia. Initial cystoscopy identified the opening of the PUC at the midline of the verumontanum. The verumontanum, which led to the PUC cavity, was cannulated using a French 5 open-ended catheter. A pediatric cystoscope was inserted to directly visualize and assess the cyst and its contents which were mildly filled with sediments. An open-ended catheter was placed within the PUC to facilitate intraabdominal identification (Fig. 2, Fig. 3). Other studies on the excision of prostatic utricle cysts described leaving a cystoscope within the cyst because the light at the end of the cystoscope served as a visual guide to its location as well as a way to manipulate the cyst intra-abdominally during excision. The 12 mm robotic camera port was inserted in the supraumbilical area, while two other ports were placed in each hemiabdomen to form a W configuration. A transabdominal posterior approach to peritoneal dissection was used to expose seminal vesicles and vas deferens. The bilateral vas deferens were carefully identified and were laterally inserted into the apex of the PUC. This was also noted in two of our previous cases done laparoscopically. Additionally, because of its insertion, the vas deferens must be ligated to facilitate excision. The PUC was circumferentially dissected and freed. Caudal dissection of the base of the PUC eventually led to its insertion into the urethra. The PUC was unroofed, revealing the previously inserted open ended catheter during cystoscopy, and was subsequently excised (Fig. 4). Careful dissection was ensured to leave at least a centimeter of the cyst to prevent injury and distortion of the urethra and bladder neck. The defect was repaired with continuous Vicryl sutures. The excised specimen was delivered using a camera port (Fig. 5). An indwelling Foley catheter was left in place after the procedure. Subsequently, laparoscopic ports were closed. The operation lasted 122 min with an estimated blood loss of 20 ml (Supplementary Video).

In the next phase of the procedure, microdissection testicular sperm extraction was performed under microscopy. Viable, motile sperm cells were harvested, adequate for five to six *in vitro* fertilization (IVF) cycles. Biopsy of both testis was done revealing normal spermatogenesis and maturation in all seminiferous tubules which suggests obstructive azoospermia from the PUC causing the patient's infertility. The postoperative clinical course was uneventful and the patient was discharged on the second postoperative day with the Foley catheter in place. Patient followed up after a week and the catheter was subsequently removed. Biopsy findings of the excised PUC also showed consistent findings of benign prostatic utricle.

Incubation of sperm and subsequent fertilization of eggs *in vitro* produced five embryos ready for implantation into the uterus. Upon consultation, the patient and his female partner attempted a trial *in vitro* fertilization last October 2023 and were able to have a successful pregnancy to a healthy term baby boy who was born in July 2024.

## 3 Discussion

Cyst formation in the lower genitourinary tract is a rare encounter and can be differentiated with respect to the prostate gland- intraprostatic and extraprostatic cysts. PUCs, which are embryological remnants of the Müllerian duct communicating with the urethra, is a type of intraprostatic cyst. The lack or dysfunction of inhibiting factors of the Müllerian duct results in incomplete regression of this duct creating a pocket where urine can stagnate predisposing patients to recurrent urinary tract infections (UTIs) and irritation. Recurrent inflammation leads to strictures that would then lead to obstructive and irritative urinary symptoms.

Presentations can vary depending on the size of the PUC as well as the age of the patient. As of writing, there is no known PUC size where patients are expected to be symptomatic; however, a study suggests that a PUC size of about 2.5 cm or greater would lead patients to feel uncomfortable [5]. Presentations also vary among pediatric and adult male patients. Pediatric patients often present with recurrent UTIs as often as six times annually [9]. Similar findings were also observed from pediatric cases of PUCs presenting as UTI with higher frequency among boys with hypospadias or those with genital anomaly [6,7,10,11]. On the other hand, adult cases are rare as those with PUCs are addressed at an earlier age. Moreover, there is limited literature on PUCs among adult male patients. Saito and colleagues [12] presented a married couple with difficulty conceiving for 10 months. The patient had been asymptomatic and physical examination was also unremarkable. However, work-up determined obstructive azoospermia secondary to PUC. Similar findings were observed in our report. This highlights the varying presentation of PUC complications across age groups. Those diagnosed early presents symptomatically whereas adult male patients are asymptomatic but present with more permanent complications.

The patient's infertility is primarily secondary to the anatomical obstruction of the vasal channel from the PUC. Moreover, this is aggravated by recurring infection and inflammation from the retrograde influx of urine remaining in the cyst. Chronic stress causes strictures within the vas deferens and urethra further obstructing the flow of sperm cells and urine altogether. These processes explain the voiding difficulties and infertility the case patient is experiencing. Obstructive azoospermia is confirmed *via* intraoperative testicular sperm extraction where sperm cells are obtained. Since there are cases where the vas deferens is attached to the PUC [12], ligation and reconstruction may be done intraoperatively which can lead to male azoospermia. In these cases, sperm banking should be offered.

Infertility is a serious consequence of PUC warranting not only PUC excision but also, sperm extraction to aid *in vitro* fertilization. This emphasizes a good clinical eye in identifying patients presenting with PUC complications. Diagnosis is primarily made by imaging such as cystourethroscopy, ultrasonography, or magnetic resonance imaging [11]. Suspicion for PUC should be raised among patients presenting with intersex disorders, hypospadias, and recurrent UTIs or epididymitis. On ultrasonography, PUC can present as a midline dilated anechoic cystic structure posterior to the urethra and the urinary bladder. On magnetic resonance, its cystic character is more appreciated on T2-weighted imaging (Fig. 1). Fluoroscopic guidance can also detect PUC but is rarely done among adult patients. This method also relies on cavity filling where incomplete filling may render a false negative result. Lastly, cystourethroscopy provides better visualization of the posterior urethra and assess cavity extent which may be significant in the surgical approach.

There is no consensus for surgical management for PUC. The limited cases among adult patients and level of difficulty poses a challenge in creating a standard surgical approach to PUC excision. Open surgery had been the first approach which can range from transperitoneal, perineal, anterior, posterior, or sagittal transrectal techniques [9]. Higher risk of complication is expected as open surgeries require extensive pelvic dissection to expose the operative field and locate the PUC for resection. In fact, a 2016 study comparing open transvesical technique *versus* laparoscopic approach showed safer, minimally invasive, and better outcomes in the latter approach. This is further supported by a 2020 study proving laparoscopy provides good visual exposure making dissection more precise thereby improving cosmesis [9]. However, newer and more innovative technology is making its way in the surgical field with robotics surgery setting the stage for 3D imaging and better magnification. This enables surgeons to see better and make better surgical choices. It removes assistive maneuvers in laparoscopy such as transabdominal hitch stitches to elevate the pelvic structures and cystoscopy guidance for manipulation of the PUC [13].

## 4 Conclusions

PUCs are rare congenital disorders in males from incomplete regression and obliteration of the Müllerian duct system. Most diagnoses are made at a young age, rarely reaching adulthood and PUC complications. However, this case report presents a 29-year old male with a 10-year history of epididymo-orchitis and complains of voiding difficulties and infertility. Work-up determined a large PUC causing obstructive azoospermia. Surgical management was warranted and robotic excision was done with testicular sperm extraction for plans of *in vitro* fertilization. There is no consensus on the standard surgical modality for PUCs but it has been shown that robotics surgery provided better exposure of the operating field thereby increasing surgical precision of the performing surgeon. Such an approach was shown to have less intraoperative complications and better postoperative outcomes. On follow-up, the patient denied any irritative or obstructive urinary symptoms and confided the success of bearing a healthy baby boy after one attempt of *in vitro* fertilization.

## Consent

The patient has been provided with adequate information regarding the publication of this case reports, its implications and potential audience. The patient has understood the purpose and authorizes the use of relevant information while maintaining confidentiality to the extent possible.

Written informed consent was obtained from the patient for publication and any accompanying images. A copy of the written consent is available for review by the Editor-in-Chief of this journal on request.

## Ethical approval

This study was granted a letter of exemption by the Institutional Ethics Review Committee Research and Biotechnology Group of St. Luke's Medical Center, Global City, Philippines (Reference No. SL – 24289).

## Funding

No funding was received for this study.

## Author contribution

• Conceptualization - NM
• Data curation – N/A
• Formal analysis – N/A
• Funding acquisition – N/A
• Investigation – NM, DS, MC
• Methodology – NM, DS
• Project administration – NM, MC
• Resources – N/A
• Software – N/A
• Supervision - NM
• Validation – NM, DS, MC
• Visualization – NM, MC
• Roles/Writing – original draft - MC
• Writing – reviewing & editing – NM, MC

## Guarantor

Nikko J. Magsanoc, M.D.

Dennis P. Serrano, M.D.

Michelangelo S. Cobangbang, M.D.

## Declaration of competing interest

The authors hereby declare that they have no affiliations with or involvement in any organization or entity in the subject matter or materials discussed in this manuscript.

## Appendix A Supplementary data

Supplementary Video Image 1

## References

[1] Lotti F., Corona G., Cocci A., Cipriani S., Baldi E., Degl’Innocenti S., Franco P.N., Gacci M., Maggi M. (2018). The prevalence of midline prostatic cysts and the relationship between cyst size and semen parameters among infertile and fertile men. Hum. Reprod..

[2] Goruppi I., Avolio L., Romano P., Raffaele A., Pelizzo G. (2015). Robotic-assisted surgery for excision of an enlarged prostatic utricle. Int. J. Surg. Case Rep..

[3] Jia W., Liu G.C., Zhang L.Y., Wen Y.Q., Fu W., Hu J.H., Xia H.M. (2016). Comparison of laparoscopic excision versus open transvesical excision for symptomatic prostatic utricle in children. J. Pediatr. Surg..

[4] Mostafa I.A., Woodward M.N., Shalaby M.S. (2018). Cystoscopic-assisted laparoscopic excision of prostatic utricle. J. Pediatr. Urol..

[5] Qiu Y., Liu Y., Ren W., Ren J. (2018). Prostatic cyst in general practice: a case report and literature review. Medicine (Baltimore).

[6] Macedo A., Di Migueli Del Debbio, R, Ottoni SL, Leal da Cruz M, Manzano JP. (2020). Robotic-assisted excision of a prostatic utricle cyst in a 12-month boy with proximal hypospadia and 45X0/ 46XY karyotype. J. Pediatr. Urol..

[7] Nguyen A., Arora H., Reese J., Kaouk J., Rhee A. (2018). Robot-assisted laparoscopic excision of prostatic utricle in a 3-year old. J. Pediatr. Urol..

[8] Sohrabi C., Mathew G., Maria N., Kerwan A., Franchi T., Agha R.A. (2023). The SCARE 2023 guideline: updating consensus surgical CAse REport (SCARE) guidelines. Int J Surg Lond Engl..

[9] Boybeyi-Turer O., Demirbilek H., Soyer T. (2020). Cystoscopy-guided laparoscopic excision of prostatic utricle: report of a case. European J Pediatr Surg Rep..

[10] Priyadarshi V., Singh J.P., Mishra S., Vijay M.K., Pal D.K., Kundu A.K. (2013). APSP J Case Rep..

[11] Shakir R., Packer M.G., Balsara Z.R. (2021). An enlarged and infected prostatic utricle as a rare cause of lower urinary tract symptoms in adolescent males. Case Rep Pediatr..

[12] Saito T., Komeya M., Usui K., Kuroda S., Takeshima T., Takashima K., Ikeda M., Kondo Y., Yumura Y. (2022). A case of obstructive azoospermia secondary to genitourinary tract infection caused by a prostatic utricle cyst. Asian J. Androl..

[13] Bayne A.P., Austin J.C., Seideman C.A. (2021). Robotic assisted retrovesical approach to prostatic utricle excision and other complex pelvic pathology in children is safe and feasible. J. Pediatr. Urol..

